# Soil warming increases soil temperature sensitivity in subtropical Forests of SW China

**DOI:** 10.7717/peerj.7721

**Published:** 2019-09-25

**Authors:** Chaoxiang Yuan, Guiqing Zhu, Shuangna Yang, Gang Xu, Yingyun Li, Hede Gong, Chuansheng Wu

**Affiliations:** 1 School of Geography and Ecotourism, Southwest Forestry University, Kunming, China; 2 Anhui Province Key Laboratory of Environmental Hormone and Reproduction, Anhui Province Key Laboratory of Embryo Development and Reproductive Regulation, Fuyang Normal University, Fuyang, China

**Keywords:** Global warming, Soil carbon efflux, Soil water sensitivity coefficient

## Abstract

**Background:**

Soil respiration (*R*_*S*_) plays an important role in the concentration of atmospheric CO_2_ and thus in global climate patterns. Due to the feedback between *R*_*S*_ and climate, it is important to investigate *R*_*S*_ responses to climate warming.

**Methods:**

A soil warming experiment was conducted to explore *R*_*S*_ responses and temperature sensitivity (*Q*_10_) to climate warming in subtropical forests in Southwestern China, and infrared radiators were used to simulate climate warming.

**Results:**

Warming treatment increased the soil temperature and *R*_*S*_ value by 1.4 °C and 7.3%, respectively, and decreased the soil water level by 4.2% (%/%). Both one- and two-factor regressions showed that warming increased the *Q*_10_ values by 89.1% and 67.4%, respectively. The effects of water on *Q*_10_show a parabolic relationship to the soil water sensitivity coefficient. Both *R*_*S*_ and *Q*_10_ show no acclimation to climate warming, suggesting that global warming will accelerate soil carbon release.

## Introduction

Soil is the largest pool of organic carbon in the terrestrial ecosystem, playing a crucial role in the global carbon cycle. *R*_*S*_ serves as a significant source of atmospheric CO_2_ (Friedlingstein et al., 2006; Bond-Lamberty & Thomson, 2010) and is regulated by temperature (Giardina et al., 2014). Therefore, slight changes in *R*_*S*_ due to global warming will increase levels of atmospheric CO_2_ and thereby be feed back into the global climate system.

Numerous studies have shown that *R*_*S*_ rates typically increase over the course of short-term soil warming experiments (Gonzalez-Meler et al., 2017; Xue & Tang, 2018; Zhao et al., 2018) and long-term warming experiments (Noh et al., 2016; Zhou et al., 2016) and likely due to the promotion of soil microbial activity and a lack of restrictions on soil organic matter during warming treatment periods. Some studies also report that warming decreases *R*_*S*_ levels during long-term warming experiments (Melillo et al., 2002; Marañón Jiménez et al., 2017; García-Palacios et al., 2018) and mainly due to the excessive consumption of carbon pools. Some studies show that short-term warming treatment can also decrease *R*_*S*_ levels (Schindlbacher et al., 2012; Liu et al., 2016) due to droughts caused by climate warming. Warming treatment also has no significant effect on *R*_*S*_ (Wang et al., 2017). Therefore, *R*_*S*_ responses to warming remain unclear and must be studied further.

The *Q*_10_ of *R*_*S*_ is an important parameter of how *R*_*S*_ responds to warming. Some studies show that warming treatment reduces the *Q*_10_ value (Boone et al., 1998; Luo et al., 2001; Wang et al., 2016; Noh et al., 2017) due to soil microorganism adaptation to warming. This adaptation may be due to a decrease in soil microbial carbon utilization and enzyme activity (Allison, Wallenstein & Bradford, 2010) and to changes in soil microbial community structures (Ziegler et al., 2013). Simultaneously, the *Q*_10_ level exhibits no adaptive response to climate warming (Reth et al., 2009) and even increases under warming conditions (Cao et al., 2018; Zhao et al., 2018).

Acclimatization has been found to decrease for *R*_*S*_ and *Q*_10_ under warming conditions (Luo et al., 2001; Melillo et al., 2002). However, a previous study shows that heterotrophic respiration in subtropical forests of Southwestern China show no acclimation to warming (Wu et al., 2016). While it is important to identify effects of warming on subtropical forests (Wu et al., 2016), *R*_*S*_ and *Q*_10_ responses to warming in such forests remain unclear. We hypothesized that *R*_*S*_ and *Q*_10_ show no acclimation to warming in the forests. To test this hypothesis, we conducted control and warming experiments on subtropical forests of the Ailao Mountains to identify the effects of warming on *R*_*S*_ and *Q*_10_ levels in these forests.

## Materials & Methods

### Site description

The experimental field is located at site of the Ailaoshan Station for Subtropical Forest Ecosystem Studies (24°32′N, 101°01′E; 2,480 m above sea level) of the Chinese Ecological Research Network. According to monitoring data for 2002 to 2011, the annual average air temperature was 11.3 °C; the maximum air temperature was 15.6 °C (July); the minimum temperature was 5.7 °C (January); the annual average precipitation level was 1,778 mm, and rainfall levels reached close to 85% of total annual rainfall for the rainy season (May–October) (Wu et al., 2014). The main type of soil found in the area yellowish-brown with a pH value of 4.5; soil organic carbon levels reach 303.8 g kg^−1^ and total nitrogen levels reach 18.38 g kg^−1^ in the humus horizon (Chan et al., 2006). The main species found in the area include *Lithocarpus hancei, Castanopsis wattii, Schima noronhae*, and *Lithocarpus xylocarpus* (Wu et al., 2014).

### Experimental design

In the subtropical forest, six plots of 1 m × 1 m were randomly selected, and two treatments were established, including control and warming treatments with three replicates. A carbonizing infrared radiator was applied as the heat radiation source for the warming treatment, and a 200-watt carbon infrared radiant heating lamp (each lamp was 90 cm long and each lampshade was 100 cm long, 15 cm wide, and 10 cm deep) was placed approximately 1.0 m above the sample side of each plot. A continuous heating mode was adopted from June 2016 to May 2017, supplying continuous power except during power outages. In each plot, a PVC pipe (inner diameter of 20 cm and soil depth of two cm) was placed to directly couple with an external PVC pipe (outer diameter of 20 cm, upper seal base opening and height of 20 cm) for further measurements of *R*_*S*_.

### Data collection and calculations

Soil CO_2_ efflux was measured monthly using a gas analyser (Li-820; Li-Cor, Lincoln, NE, USA) between 9:00 and 11:00 AM (Beijing Time) to measure *R*_*S*_ levels. Soil temperatures (*T*, °C) were measured with a digital thermometer (6310; Spectrum, Illinois, USA), and soil water content (*W*, %) was measured via time domain reflectometry (MP-KIT; Beijing Channel, Beijing, China) at a depth of 50 mm. The *R*_*S*_ rate was calculated as follows:

(1) \begin{eqnarray*}& & {R}_{S}= \frac{V\cdot P}{R\cdot S\cdot Ta} \cdot \frac{dc}{dt} \end{eqnarray*}

where *R*_*S*_ is the soil CO_2_ efflux value (µmol m^−2^ s^−1^); *V* is the chamber volume (m^3^); *S* is the chamber area (m^2^); *R* is the gas constant (8.314 Pa m^3^ k^−1^ mol^−1^); *T* a is air temperature (K); *P* is air pressure (Pa), and *dc/dt* is the slope of variations in CO_2_ concentrations overtime for the measurement period.

We used a one-factor regression to determine the relationship between *R*_*S*_ and *T* and a two-factor regression to determine relationships of *R*_*S*_ with *T* and *W*. The regression equations applied are as follows (Xu & Qi, 2001a; Liu et al., 2018):

(2) \begin{eqnarray*}& & {R}_{S}=a\cdot {e}^{bT}\end{eqnarray*}

(3) \begin{eqnarray*}& & {R}_{S}=a\cdot {e}^{bT}\cdot {W}^{c}.\end{eqnarray*}

Where *a*, *b* and *c* are estimated from the regressions; *b* is the soil *Q*_10_ coefficient, and *c* is the soil water sensitivity coefficient.

*Q*_10_ is calculated using the following equation (Luo et al., 2001):

(4) \begin{eqnarray*}& & {Q}_{10}={e}^{10b}.\end{eqnarray*}

Where *b* is derived from Eqs. (2) and (3).

Different *Q*_10_ values derived from the one- and two-factor regressions are attributable to soil water effects, and the effect on *Q*_10_ (*WE*_*Q*_10__) was calculated with the following equation:

(5) \begin{eqnarray*}& & W{E}_{Q10}= \frac{{Q}_{10-\mathrm{one}-\text{factor}}-{Q}_{10-\mathrm{two}-\text{factor}}}{{Q}_{10-\mathrm{two}-\text{factor}}} \times 100\text{%}\end{eqnarray*}

The parameters were estimated from the models listed above using the nonlinear regression dynamic fit wizard, and t tests were used to test differences in the *R*_*S*_, *T*, *W*, and *Q*_10_ values of the control and warming treatments (Version 12; Systat Software, Inc., Point Richmond, California, USA).

## Results

### Effect of warming on soil environment factors

**Figure 1:**
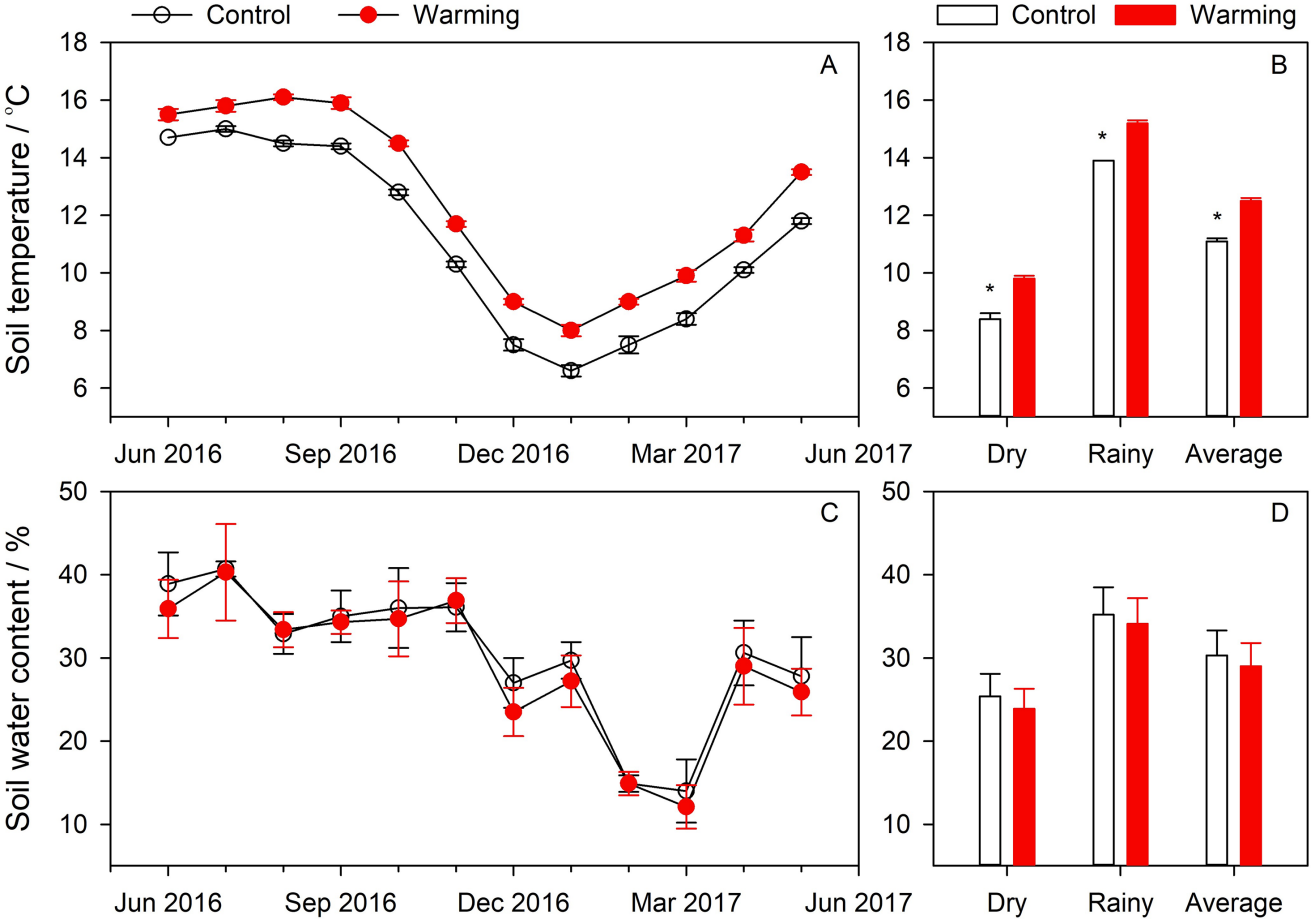
Seasonal variations of soil temperature (A) and soil water content (C), and comparisons of soil temperature (B) and soil water content (D) between control and warming treatment.

The warming treatment increased the soil temperature but did not change seasonal variation patterns (Fig. 1A). Mean annual soil temperatures measured from the control and warming treatments were recorded as 11.1 ± 0.1 °C and 12.5 ± 0.1 °C, respectively, and warming significantly increased the soil temperature by 1.4 °C (Fig. 1B). During the measurement period, the warming treatment significantly affected soil temperatures during both the dry and rainy seasons (Fig. 1B). However, warming did not change soil water content levels, and in terms of seasonal variations, we found no significant differences between dry season, rainy season and yearly trends (Figs. 1C & 1D). Mean annual soil water levels measured from the control and warming treatments were recorded as 30.3 ± 3.0% (v/v) and 29.0 ± 2.8% (v/v), respectively, reduced by 4.2% (%/%) (Fig. 1D).

### Effect of warming on soil respiration

**Figure 2:**
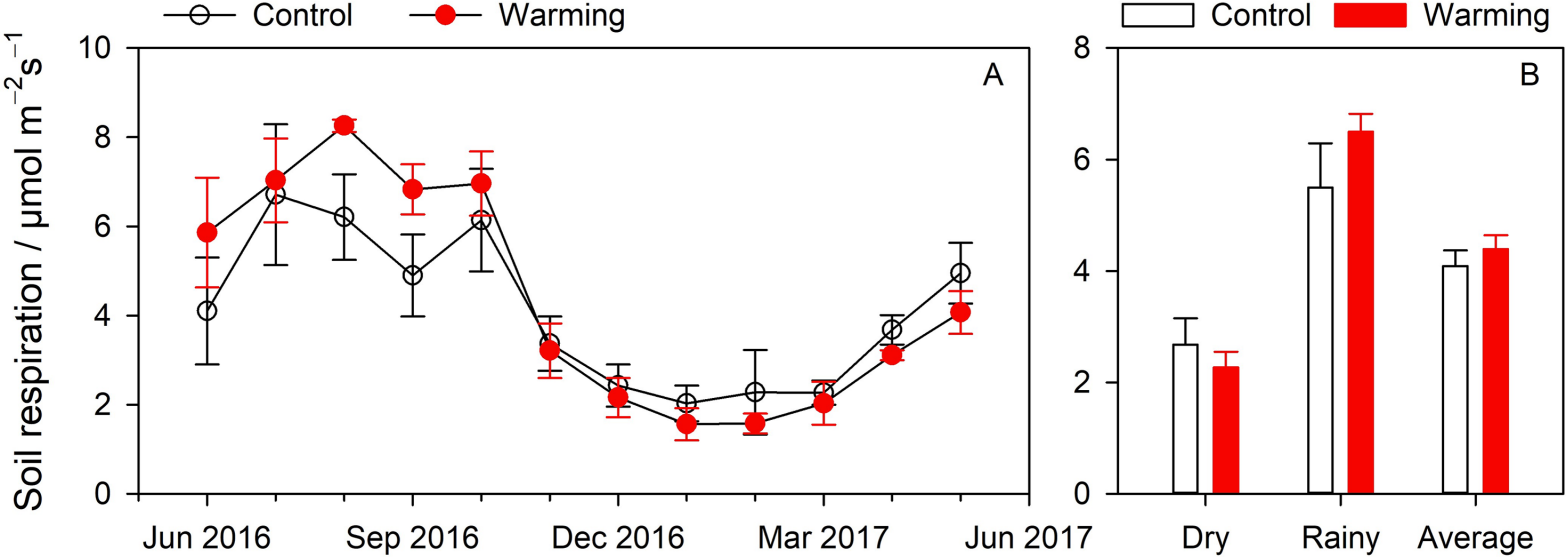
Seasonal variations (A) and comparison (B) of soil respiration between control and warming treatment.

Soil warming increased *R*_*S*_ levels in the rainy season but suppressed them in the dry season (Fig. 2A). No significant differences in means of the control and warming treatments were observed across dry season, rainy season, and annual period (Fig. 2B). In the dry season, mean values of the two treatments were recorded as 2.68 ± 0.47 µmol m^−2^s^−1^ and 2.27 ± 0.28 µmol m^−2^s^−1^, respectively, with the warming treatments showing a reduction of 15.0%. In the rainy season, mean values for the two treatments were recorded as 5.50 ± 0.79 µmol m^−2^s^−1^ and 6.50 ± 0.32 µmol m^−2^s^−1^, respectively, with the warming treatments showing an increase of 18.1%. The mean annual *R*_*S*_ for the two treatments was recorded as 4.09 ± 0.28 µmol m^−2^s^−1^ and 4.39 ± 0.25 µmol m^−2^s^−1^ where values increased by 7.3% during the warming treatment.

### Effect of warming on temperature sensitivity

**Figure 3:**
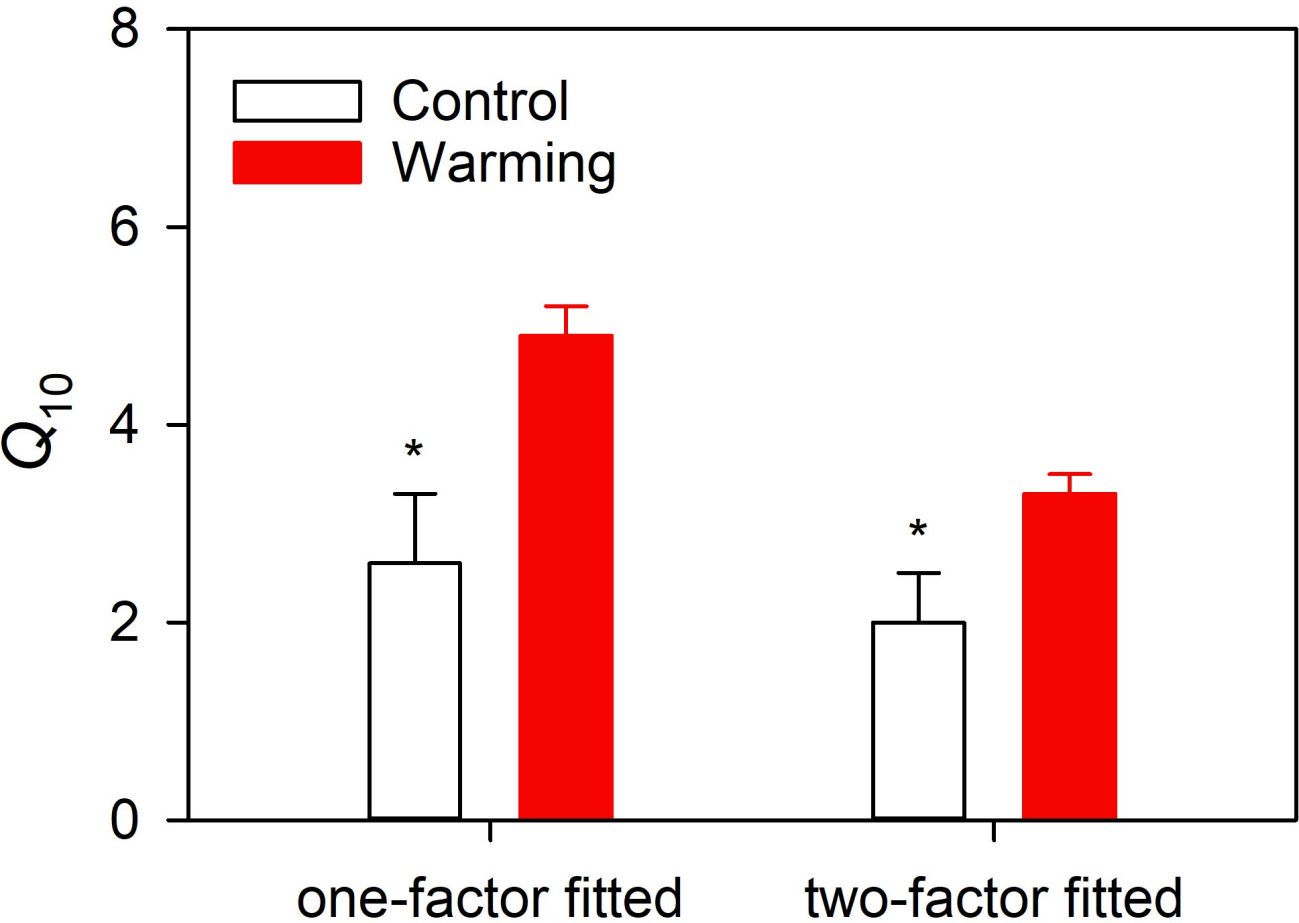
Comparison of temperature sensitivity between control and warming treatment.

**Figure 4:**
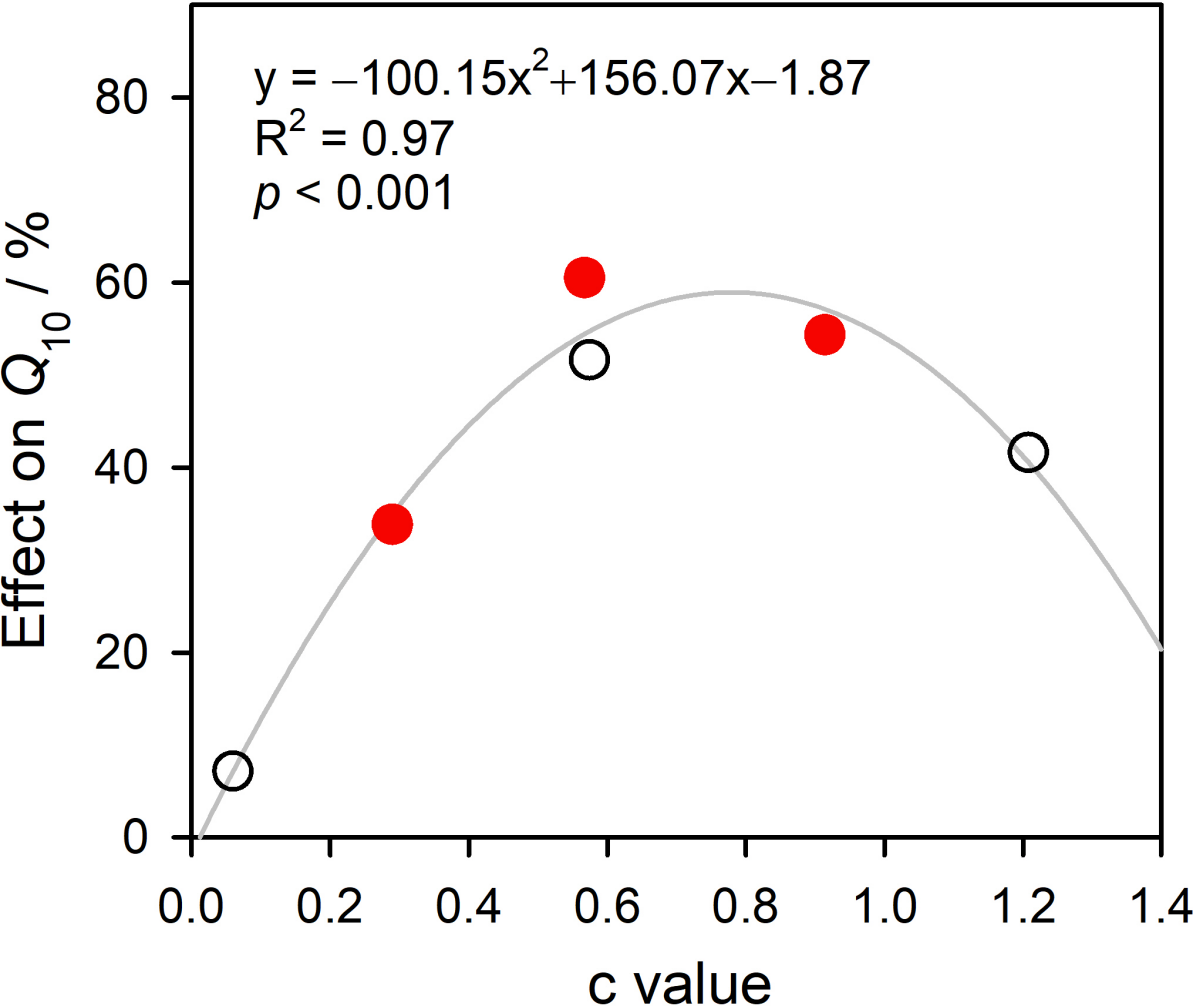
Relationship between soil water sensitivity and effect of soil water on temperature sensitivity.

From one-factor regression model results, the *Q*_10_ values of the control and warming treatments were recorded as 2.6 ± 0.7 and 4.9 ± 0.3, respectively, and warming significantly increased the *Q*_10_ value by 89.1%. According to two-factor regression model results, the *Q*_10_ values reached 2.0 ± 0.5 and 3.3 ± 0.2, respectively, and warming significantly increased the *Q*_10_ values by 67.4% (Fig. 3). One- and two-factor regression parameters are given in Table 1. *WEQ*_10_ increased when the c values fell below 0.779 and decreased when the *c* values exceeded 0.779 (Fig. 4).

**Table 1:** Parameters of one-factor and two-factor regressions.

Treatments	*R*_*S*_ = *ae*^*bT*^*W*^*c*^					*R*_*S*_ = *ae*^*bT*^			
	*a*	*b*	*c*	*R*^2^	*p*	*a*	*b*	*R*^2^	*p*
Control	0.0197	0.0864	1.2078	0.86	<0.001	0.9823	0.1212	0.61	<0.001
	0.3139	0.0369	0.5743	0.55	<0.001	1.5024	0.0785	0.48	<0.001
	1.4718	0.0724	0.0599	0.62	<0.001	1.6548	0.0793	0.62	<0.001
Warming	0.0370	0.1119	0.9139	0.96	<0.001	0.5697	0.1553	0.78	<0.001
	0.1080	0.1191	0.5672	0.84	<0.001	0.4224	0.1664	0.80	<0.001
	0.3484	0.1251	0.2896	0.89	<0.001	0.6023	0.1542	0.87	<0.001

## Discussion

Warming treatment can effectively increase soil temperatures (Wang et al., 2019; Li et al., 2018) and thereby decrease soil water levels (Wang et al., 2014; Chen et al., 2018). In our experiment, warming treatment resulted in higher soil temperatures than those observed in the control treatment (Figs. 1A & 1B), and the soil water levels were found to be lower than those of the control treatment (Figs. 1C & 1D). A number of previous studies show that warming experiments significantly increase *R*_*S*_ levels (Zheng et al., 2009; Zhao et al., 2018; Zou et al., 2018). In our experiment, the warming treatment resulted in an increase in the average *R*_*S*_ by 7.3% due to soil warming of 1.4 °C. These findings are less significant than others reported. For instance, *R*_*S*_ has been found to increase by 32% with 4 °C of warming (Noh et al., 2016), by 28% with 2.5 °C of warming (Bamminger, Poll & Marhan, 2018), and even by 7.1% with 0.47 °C of warming (Xia et al., 2009). Wu et al. (2016) found the warming effects on soil carbon flux to be positively related to the warming effects on soil temperature. The warming effects on soil temperature observed in the present study are less significant (or stronger) than those reported above, leading to lesser (or stronger) warming effects on *R*_*S*_. In the present study, warming treatment was found to have no significant effect on *R*_*S*_, agreeing with the results found for another subtropical forest in Guangxi, China (Wang et al., 2017). However, 1.7 °C warmer temperatures significantly increase heterotrophic respiration in subtropical forests in Guangxi, China, corroborating results reported for our study region (Wu et al., 2016). Therefore, our results showing no significant effects are likely attributable to the fact that warming suppresses autotrophic respiration (Wang et al., 2017).

We also found the warming treatment to have different effects during the dry and rainy seasons (Figs. 2A and 2B). The study area is located in a monsoon region where temperature and humidity levels are synchronized and where *R*_*S*_ is affected by effects of interactions between soil temperature and soil water content (Table 1). In the rainy season, soil water levels were high while decreases in soil water content due to warming were limited (3.2%, %/%). Therefore, soil water content had no restrictive effects. However, in the dry season, soil water levels and soil temperatures were both low and positive effects of soil warming were less significant than negative effects of soil water content due to warming, as soil drought due to warming can offset warming effects (Schindlbacher et al., 2012). Therefore, *R*_*S*_ levels were found to be lower under the warming treatment than under the control treatment in the dry season.

While warming has been widely shown to decrease the *Q*_10_ values (Luo et al., 2001; Li et al., 2016; Noh et al., 2017), we found warming to increase *Q*_10_ values consistent with previous studies (Teramoto et al., 2018; Zhao et al., 2018). Warming treatment decreased basal respiration in the dry season and increased respiration in the rainy season, thereby increasing seasonal amplitudes (Fig. 2). However, warming did not change seasonal amplitudes of soil temperature (Fig. 1A), leading to higher *Q*_10_ values from regression models. Soil is intrinsically sensitive to temperature as determined by substrate properties and initial temperature conditions (Davidson & Janssens, 2006). *Q*_10_ levels of *R*_*S*_ calculated from equation models show *Q*_10_ affected by intrinsic *Q*_10_ and environmental factors (e.g., soil water conditions) (Davidson & Janssens, 2006). *R*_*S*_ is directly influenced by substrate supplies, soil temperatures, and effects of soil temperature and soil water content on substrate diffusion and availability (Davidson, Janssens & Luo, 2006). Therefore, both soil temperature and soil water content affect *Q*_10_ values, explaining 93% of seasonal variations in *Q*_10_ (Xu & Qi, 2001b).

In this study, two models (Eqs. (2) and (3)) were used to reflect the relationship between *R*_*S*_ and soil temperature and to calculate the *Q*_10_ values. GLM analysis suggests that both the treatment and model had significant effects on the *Q*_10_ values (Table 2). The one-factor model only considers relationship between *R*_*S*_ and soil temperature, while the two-factor model considers the effects of both soil temperature and soil water content on *R*_*S*_. As the *Q*_10_values were calculated from these models, any factors affecting *R*_*S*_ would affect them. Therefore, soil water availability affects *Q*_10_ (Zhong, Shen & Fu, 2016). The *Q*_10_ values derived from the one-factor model are significantly higher than those derived from the two-factor model, as the *Q*_10_ value calculated from the one-factor model covers soil water effects. In comparing the two models, we measured the effects of water on *Q*_10_ (*WE*_*Q*_10__) and found the relationship between *WE*_*Q*_10__ and the soil water sensitivity coefficient (*c* value in Eq. (3)) (Fig. 4). *WE*_*Q*_10__ exhibits a parabolic regression relationship with the *c* value (peak value of 0.779).

**Table 2:** Result of GLM analysis on temperature sensitivity.

Source	*t* value	*p* value
Treatment	5.961	<0.01
Method	−3.664	<0.01

Soil respiration is driven by soil microbial rhizosphere activities and is affected by soil temperature and the soil water content. Therefore, warming effects on *R*_*S*_ and *Q*_10_ are affected by the above factors. Warming treatment can change substrate supplies and microbial activities or communities (Wang et al., 2019; Li et al., 2018). Therefore, soil aggregate protection and microbial communities can also affect the *Q*_10_ values (Qin et al., 2019). In the present study, we focused on warming effects on *R*_*S*_ and *Q*_10_. More studies on soil properties and microbial communities should be conducted.

## Conclusions

In summary, our warming experiment increased the soil temperature by 1.4 °C, decreased the soil water level by 4.2% (%/%), and increased the *R*_*S*_ value by 7.3%. Both one- and two-factor regressions show that warming increased the *Q*_10_ values by 89.1% and 67.4%, respectively. Both *R*_*S*_ and its *Q*_10_ show no acclimation to climate warming, suggesting that global warming will accelerate soil carbon release.

##  Supplemental Information

10.7717/peerj.7721/supp-1 Data S1 Raw data

## Additional Information and Declarations

### Data Availability

The following information was supplied regarding data availability: The raw measurements are available in Data S1.
